# The Elecsys® Anti-SARS-CoV-2 and Elecsys® Anti-SARS-CoV-2 S antibody assays: Differentiating between vaccination and infection, and assessing long-term performance

**DOI:** 10.1371/journal.pone.0305613

**Published:** 2024-07-18

**Authors:** Franke A. Quee, Boris M. Hogema, Michel Molier, Ed Slot, Katja van den Hurk, Hans L. Zaaijer

**Affiliations:** 1 Department of Donor Medicine Research, Sanquin Research, Amsterdam, The Netherlands; 2 Department of Virology, Sanquin Diagnostic Services, Amsterdam, The Netherlands; 3 Department of Medical Affairs, Sanquin Corporate Staff, Sanquin Research, Amsterdam, The Netherlands; 4 Department of Clinical Virology, Amsterdam University Medical Centre, Amsterdam, The Netherlands; University of Florence: Universita degli Studi di Firenze and University of Palermo: Universita degli Studi di Palermo, ITALY

## Abstract

**Introduction:**

Serological surveillance is useful for assessing SARS-CoV-2 immunity in populations. To effectively study the presence and persistence of antibodies, it is necessary to distinguish between persons with past infection, and persons who only received vaccination. Knowledge of the duration of antibody persistence is essential for correct interpretation of surveillance results.

**Methods:**

Starting in April 2020, waning of SARS-CoV-2 antibodies was studied in a longitudinal cohort study of 495 SARS-CoV-2 antibody-positive Dutch blood donors, not pre-selected by PCR testing or disease severity. Additionally, in May 2021, a sample of donors representative for the Dutch population was tested for antibodies against the SARS-CoV-2 spike (S) protein, using the Wantai Ab ELISA and the Elecsys® Anti-SARS-CoV-2 S assay; and for antibodies against the nucleocapsid protein, which indicate past infection, using the Elecsys® Anti-SARS-CoV-2 assay.

**Results:**

The anti-S response in donors that were infected in April or May 2020 remained positive in 100% of donors in the Elecsys® Anti-SARS-CoV-2 S assay one year after infection, after which follow up of waning was no longer possible because of large scale vaccination. The anti-nucleocapsid response results were still positive in approximately 80% of donors two years after infection. In May 2021, 51% of the donors showed anti-S reactivity and 16.8% tested positive for anti-nucleocapsid antibodies.

**Conclusion:**

Infection with SARS-CoV-2 resulted in spike and nucleocapsid antibody levels still detectable in the majority of donors 1–2 years after infection. In May 2021, 51% of donors were vaccinated and 16.8% had had an infection. Thus, both Elecsys® SARS-CoV-2 antibody assays can be used to reliably assess the vaccination and infection status of individuals.

## Introduction

The first case of Coronavirus Disease 2019 (COVID-19), caused by Severe Acute Respiratory Syndrome Coronavirus 2 (SARS-CoV-2), in the Netherlands was notified on February 27^th^ 2020 [1]. Infection preventive measures, like physical distancing, closure of schools, shops, sport facilities and drinking and eating facilities, followed rapidly. As of June 1^st^ 2020, everyone with symptoms associated with SARS-CoV-2 infection could have a free PCR test [2]. However, this did not prevent a second wave of infections starting mid-September 2020. On January 6^th^ 2021 a nationwide vaccination campaign started which rapidly reduced the number of COVID-19 patients, until more rapidly spreading variants emerged [3, 4].

One of the most important tools for controlling the pandemic is detection of SARS-CoV-2 in individuals. Using real-time reverse transcription polymerase chain reaction (rt-PCR) based tests or rapid antigen tests, symptomatic individuals can be tested and isolated if positive. However, this approach does not capture asymptomatic individuals who did not seek testing, and it is not able to detect past infections [5]. Therefore, rt-PCR testing does not facilitate an estimation of the proportion of the population ever infected with SARS-CoV-2. Serological testing for SARS-CoV-2 antibodies can assist policy makers by providing estimations of the level of immunity of the population; the proportion with past infection or vaccination only; the persistence and levels of antibodies; and the occurrence of re-infections.

The waning of antibodies complicates the estimation of the proportion of persons with past infection, and the increasing vaccination coverage makes this even more challenging. To effectively study the humoral immune response in both individuals with infection-acquired immunity and vaccine-acquired immunity, it is necessary to be able to distinguish these two groups. Vaccines used in the Dutch COVID-19 vaccination program induce antibodies to the spike (S) protein only [6]. To discriminate between infection-induced antibodies and vaccine-induced antibodies, a test recognizing antibodies against a viral protein other than the S protein, e.g. nucleocapsid (NC) antibodies, can be employed. Rapid waning of the NC antibody response would hamper the reliability of the estimation of the number of past infections, so interpretation of results requires a thorough assessment of the rate of waning [7].

Blood donors are popular study subjects because of the wide availability of their blood samples and their repeated donations, enabling cross-sectional and longitudinal seroprevalence studies. A wide range of serological assays has been used to perform such studies [8, 9]. The assays need to be evaluated and compared to ensure meaningful results. In this study we aim to study (1) the performance of an anti-NC and anti-RBD assay, the Elecsys® Anti-SARS-CoV-2 (Elecsys® NC) and Elecsys® Anti-SARS-CoV-2 S (Elecsys® S), respectively over time; and (2) the suitability of the Elecsys® NC and Elecsys® S assays for distinguishing between vaccinated and previously infected donors.

## Methods

### Study population

By law, Sanquin Blood Supply Foundation is the only organization authorized to collect and distribute blood and blood components in the Netherlands. Dutch blood donors aged 18 to 80 years donate over 700,000 times per year on a voluntary non-remunerated basis, at 49 fixed collection sites and 85 mobile collection sites. Donor eligibility is assessed prior to donation using a donor health questionnaire, and blood pressure and capillary hemoglobin measurements. Additionally, donors fill out a mandatory “COVID-19 health check” form upon entry of a donation center, to prevent individuals with SARS-CoV-2 infection from donating. This study was reviewed and approved by the Ethics Advisory Council of Sanquin Blood Supply Foundation.

### Study design

To monitor SARS-CoV-2 infection throughout the Netherlands, Sanquin performed a large seroprevalence study in April and May 2020. All available plasma donations (>14.000 donations) collected during April 1-15^th^ and May 10-20^th^ 2020 were tested for antibody presence [10, 11]. Archived samples of positive donors were tested to check for true seroconversion using archived pre-outbreak samples. For the current study, all donors with a positive sample in April or May 2020 were followed over time by testing their subsequent donations, to check for seroreversions; and to check for re-infections (or vaccination as of February 2021), as indicated by a significant increase in antibody titers. We further refer to this cohort as the longitudinal cohort study, which was used to study the performance of the Elecsys® NC and Elecsys® S antibody assays over time.

Additionally, we performed nation-wide weekly testing for SARS-CoV-2 antibodies from June 2020 until August 2021 in at least 2000 donations each week. For the purpose of this study, from the weekly testing, we included donations collected from May 17-20^th^ 2021. We further refer to this study as the cross-sectional study. This study was used to assess the suitability of the Elecsys® NC and Elecsys® S antibody assays for distinguishing between vaccinated and previously infected donors.

### Sample selection

For the cross-sectional study, a subset of samples from all donations made between May 17-20^th^ 2021, was selected based on age, sex, and region of residence, to provide optimal coverage and representation of the general Dutch population, as follows [12]. The number of donations collected for every combination of the first three digits of the postal code of the donors’ addresses; for 10-year age groups; and for sex was calculated for the period January 1^st^ 2019 to October 11^th^ 2020. Only donations made in donation centers that are open at least once every two weeks were included to prevent large fluctuations in the number of donations from different areas. Without this correction, at certain time points, a disproportionate number of donations could originate from mobile locations that operate only a few times per year. Donations from donors that were specifically recruited for donation of SARS-CoV-2 convalescent plasma were excluded.

The number of appropriate donations to be tested (based on ideal representation of each group) for each combination of postal code, sex and age was divided by the actual number of donations for each combination, and the value obtained was used to make the selection of donations to be tested each day. For this purpose, a table containing these values for all donors was made in Excel. Each morning, a list of donor numbers from donors who made a donation the previous day, was inserted into the selection Excel file and a selection of 550 donations was made in a Monte-Carlo like fashion using the random number generator from Excel. Donors were enrolled if they were accepted for routine donation and provided consent for use of leftover samples for research. Results of the sample selection can be found in S1 Table.

### Testing for SARS-CoV-2 antibodies

To assess seroprevalence in the Dutch blood donor population, all samples were tested using the Wantai Ab ELISA (Wantai Biological Pharmacy Enterprise Co., Ltd., Beijing, China) following the manufacturer’s instructions and using a Tecan EVOlyzer (Tecan, Männedorf, Switzerland) for automated analysis. This assay employs a recombinant receptor binding domain (RBD) SARS-CoV-2 antigen of the S protein, in the double antigen sandwich format, facilitating detection of all antibody isotypes (IgA, IgM, IgG) to SARS-CoV-2. Samples tested with the Wantai Ab ELISA which had an OD/CO > 0.25 were retested. Samples with more than two-fold difference in OD/CO value, or a discrepant result between the first test and the retest, were tested once more. A discrepant result was defined as a sample that was, for example, positive in the first test, but negative in the retest. If, after retesting twice, the result was still discrepant, the final result was adjusted to positive if 2 out of 3 test results were positive and the average OD/CO value of all tests was >1. The kit lot used for screening was obtained as part of a national supply of SARS-CoV-2 kits and expired in March 2021, two months before the testing of samples for this study. To assess whether this made any difference for the final results, most of the re-testing was done with a kit lot that was produced in February 2021 and had an expiration date in 2022.

For the cross-sectional study, all samples that tested positive for SARS-CoV-2 antibodies using the Wantai Ab ELISA were additionally tested using the Elecsys® S and Elecsys® NC assays (Roche Diagnostics, Rotkreuz, Switzerland) for estimating the anti-S and anti-NC seroprevalence among Dutch blood donors, respectively [11]. The Elecsys® S is an electrochemiluminescence double-antigen sandwich immunoassay for detection of antibodies to the SARS-CoV-2 S protein RBD. The Elecsys® NC assay is an electrochemiluminescence double-antigen sandwich immunoassay for detection of antibodies to the NC protein of SARS-CoV-2. All samples were tested using the Cobas e801 machine (Roche Diagnostics, Mannheim, Germany). A sample was regarded positive for Wantai if OD/CO >1, for Elecsys® S if cutoff index (COI) > 0.8, and for Elecsys® NC if COI >1. For the longitudinal cohort study subsequent follow-up donations from donors in the cohort were periodically tested using both Elecsys® assays, and the waning of antibodies was followed until a threefold increase was detected indicative of re-infection (increase of both anti-NC and S antibodies) or vaccination (S antibody increase only).

### Statistical analysis

In both studies, donors were categorized based on antibody presence. If the donor had both S and NC antibodies, the donor was qualified as ‘past infection’. When only S antibodies were present, the donor was qualified as ‘vaccinated only’. Donors that lacked both antibodies were qualified as ‘negative’ (neither infection nor vaccination). Results of the Elecsys® S were compared to the Wantai Ab ELISA assay, to check qualitative agreement on presence of S antibodies. For studying antibody waning, the reactivity was followed in time in donors from the longitudinal cohort study. If a donor made multiple donations in a quarter, we only selected the last donation. The percentage of donors still testing positive in a quarter was calculated by dividing the number of positive samples by the number of tested samples per quarter. Waning was calculated from the first positive sample instead of the highest observed antibody titer. Donors with a missing Elecsys® S or Elecsys® NC index measurement were excluded from the analysis. Trends were estimated using General Linear Mixed Modeling (GLMM), to account for correlation within donors.

## Results

### Baseline characteristics

The longitudinal cohort study included the 495 antibody positive donors (and their follow-up samples), as detected among the 14.515 donations that were tested in April and May 2020 (Table 1). A total of 4392 donations was tested, corresponding to an average of 8.9 (range 2–25) donations per donor. For the cross-sectional study, 2189 plasma samples were tested in May 2021. For seven samples insufficient material was available for testing in all three assays, leaving 2182 samples available for this study (S1 and S2 Tables) [13]. Of these, 1049 (48%) were from female donors, and 1135 (52%) were from male donors. The majority of donors made plasma donations (51%).

**Table 1:** Study characteristics.

	Longitudinal cohort study (followed since April/May 2020)	Cross-sectional study (May 2021)	General population^$^ [13]
**Total (N, %):**	**495**	**2182**	12,615,052 (adults)
Female	250 (51%)	1048 (48%)	6,295,781 (50%)
Male	245 (49%)	1134 (52%)	6,319,271 (50%)
**Age group:**			
18–30	114 (23%)	286 (13%)	2,895,629 (23%)
31–40	64 (13%)	343 (16%)	2,129,264 (17%)
41–50	92 (19%)	412 (19%)	2,268,863 (18%)
51–60	148 (30%)	424 (19%)	2,502,394 (20%)
61–70	77 (15%)	490 (22%)	2,070,015 (16%)
>70	0 (0%)	227 (10%)	748,887 (6%)
**Donation type**			
Whole blood	28 (6%)	963 (44%)	
Plasma	434 (88%)	1068 (51%)	
Other*	33 (7%)	151 (5%)	

*specific plasma donations, such as thrombocyte apheresis, HLA-matched apheresis etc.

^$^ Aged 18–74 year old to reflect age-limits for blood donation

### Waning of S and NC antibodies

**Fig 1:**
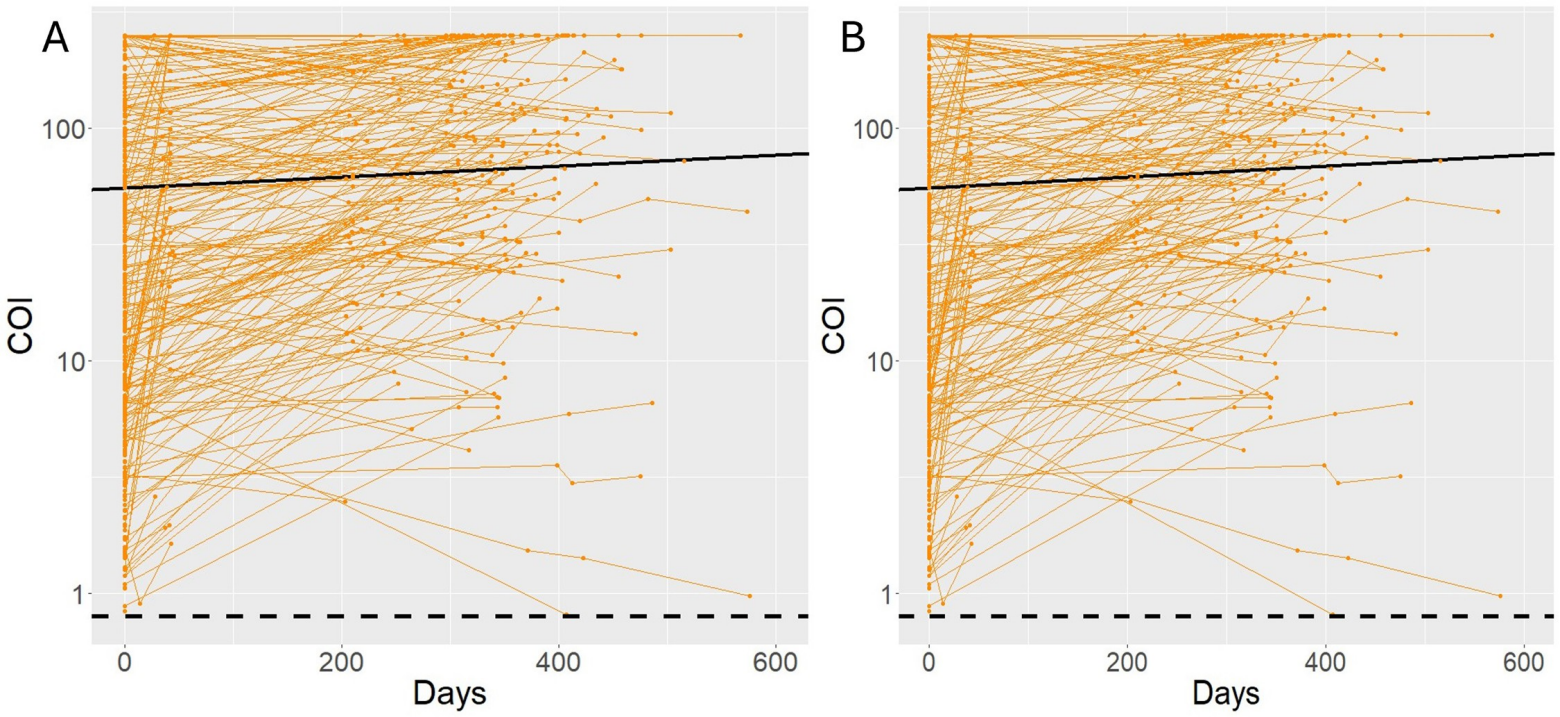
Antibody waning in (A) the Elecsys® NC and (B) Elecsys® S antibody assays. Each orange line represents serial measurements of one donor. The dotted line depicts the cut-off value of the assay. The black line is the trend line estimated using GLMM. GLMM was applied after t = 14 days for both assays.

The reliability of the estimated proportions of vaccinated and infected donors depends on consistent, persistent antibody detection in all three assays. A waning antibody response could lead to misclassification. To assess the waning of the NC and S responses, follow-up samples of donors included in the longitudinal cohort study were tested. S antibody signals in the Wantai and in the Elecsys® S assay remained stable over time, with 100% of donors still positive after 12 months in the Elecsys® S assay, and 98% in the Wantai assay (Table 2). NC antibodies as tested with the Elecsys® NC assay waned, with 87% testing positive after 12 months (Table 2) and an increasing number of donors sero-reverting, especially after one year (Fig 1A). Therefore, an alternative cutoff of COI 0.3 was used in addition to the cut-off recommended by the manufacturer. With the alternate cutoff approximately 10% more donors tested positive for NC antibodies after a 2-year period (Table 2).

**Table 2:** Proportion of positive test results over time, measured in the longitudinal cohort study (donors infected in the first months of 2020).

	Wantai	Elecsys® S	Elecsys® NC	Elecsys® NC (COI > 0.3)
**Index**	100% (495/495)	91% (385/424)	87% (426/491)	92% (454/491)
**0–3 months**	97% (304/314)	100% (44/44)	93% (53/57)	95% (54/57)
**4–6 months**	98% (260/266)	-	94% (65/69)	100% (69/69)
**7–9 months**	97% (230/238)	95% (59/65)	89% (118/133)	97% (129/133)
**10–12 months**	98% (319/324)	99% (170/171)	87% (205/236)	96% (226/236)
**13–15 months**	99% (316/320)	99% (198/200)	81% (222/275)	93% (257/275)
**16–18 months**	100% (328/329)	100% (155/155)	78% (158/202)	90% (181/202)
**19–21 months**	100% (270/270)	100% (93/93)	81% (142/176)	90% (159/176)
**22–24 months**	100% (271/271)	-	83% (144/174)	90% (156/174)

Applying GLMM, the estimated intercept and slope were respectively 1.74 and 0.000236 for the Elecsys® S assay, and 1.34 and -0.00118 for the Elecsys® NC assay (Fig 1). For visualization purposes we removed donors with a negative index sample (65 and 39 index samples for Elecsys® NC and Elecsys® S, respectively).

### Wantai vs. Elecsys® S antibody assay

**Fig 2:**
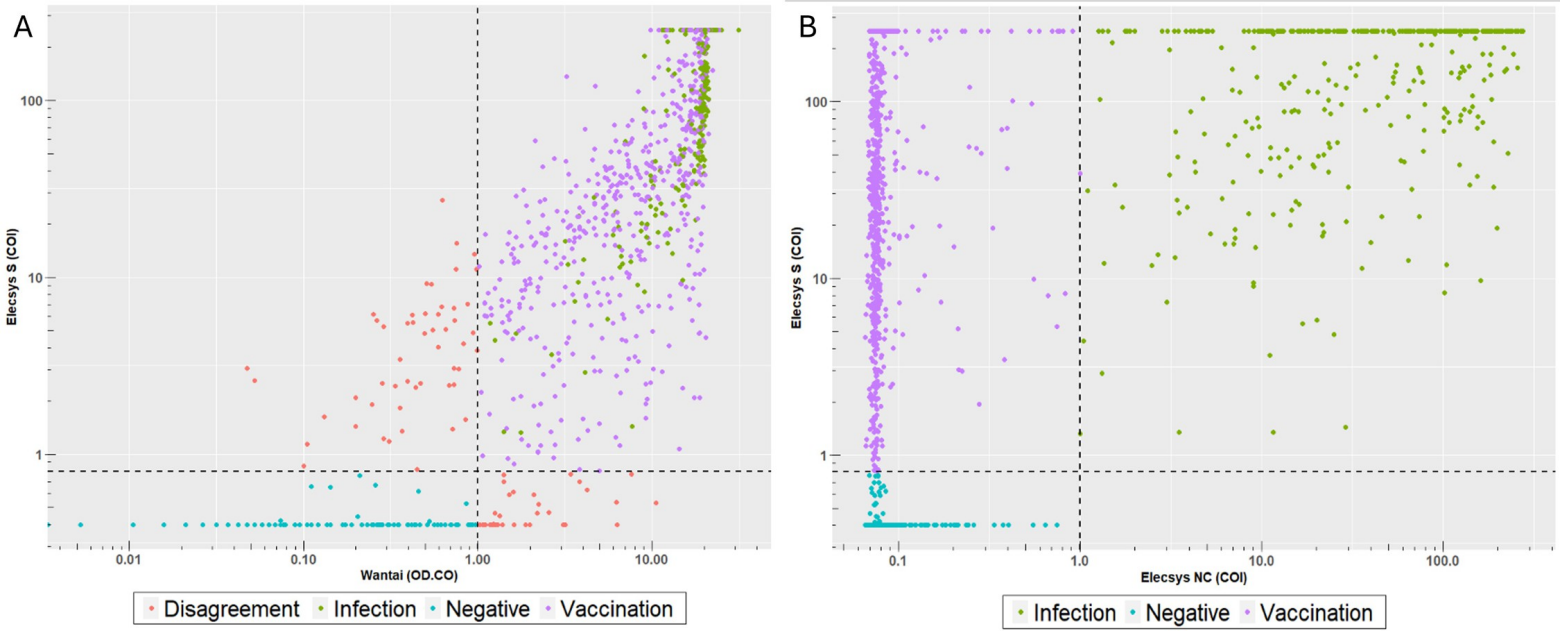
Agreement of (A) Wantai and Elecsys® S antibody assay and (B) classification of vaccinated and infected persons using the Elecsys® S and Elecsys® NC antibody assays.

In the cross-sectional study, 2182 samples of May 2021 were tested for S antibodies in the Wantai and Elecsys® S assays (Table 3 and Fig 2): 1066 (48.8%) tested positive in Wantai and 1078 (49.4%) in the Elecsys® S assay. There were few discrepant results: 40 (1.8%) tested negative in the Elecsys® S and positive in the Wantai assay, while 52 samples (2.4%) tested positive in the Elecsys® S and negative in the Wantai assay. Repeat testing of samples with an OD/CO ratio >0.25 revealed 17 qualitatively discrepant results. Fourteen of these displayed reactivity around the cutoff, explaining discrepant results; leaving only 3 truly discrepant samples (0.1%). S1 Fig displays results from the repeat-testing of the positive samples and shows overall good qualitative agreement between duplicates, irrespective of whether repeat testing was performed with a recently expired kit lot or with a recently produced lot. The qualitative agreement between the Wantai and Elecsys® S antibody assays was 95.8% (*p <* .*001*), and the majority of the discrepant results was from samples that showed reactivity above the average background in the negative test (Table 3).

**Table 3:** Qualitative agreement of Elecsys® S vs. Wantai.

		Elecsys® S result (S/CO)				
Wantai result (OD/CO)		Negative		Positive		Total
		0.4–0.5	0.5–0.8	0.8–20	20–250	
Negative	0–0.25	1018	3	8	0	1029
	0.25–1	41	4	42	1	88
Positive	1–5	25	9	142	38	214
	5–25	2	3	105	741	851
	Total	1086	19	297	780	2182

### Elecsys® NC vs. Elecsys® S antibody assay

Of 2182 tested samples, 371 samples (17.0%) were positive for presence of NC antibodies. Of these, 367 (98.9%) were also positive for S antibodies (S3 Table). However, due to the waning antibody levels, results may become negative. Therefore, the alternative cutoff of COI 0.3 was applied, which led to a slight increase of 27 (1.2%) more donors testing positive for NC antibodies [9].

### Estimated percentage of vaccinated donors

In the cross-sectional study, 16.8% of the donors tested positive for both NC and S antibodies, indicating past infection. If we include donors with isolated NC antibodies (no S antibodies), this percentage increases with 0.18 percent point, to approximately 17%. Between 32.0–32.5% of the donors tested positive for S antibodies only, thus probably acquired antibodies due to vaccination. Between 50.5–51.0% of the donors did not have any antibodies and were considered neither infected nor vaccinated. The estimates for the Elecsys® and Wantai assays were highly similar (Table 4).

**Table 4:** Distinguishing between vaccinated and infected donors.

	Elecsys® S + NC	Wantai + Elecsys® NC
**Negative**	1101 (50.5%)	1113 (51.0%)
**Infection**	367 (16.8%)	367 (16.8%)
**Vaccination**	710 (32.5%)	698 (32.0%)
**NC only**	4 (0.18%)	4 (0.18%)

## Discussion

Seroprevalence studies can provide insight in the proportion of the population that have been vaccinated against COVID and/or has been infected with SARS-CoV-2. Combining anti-SARS-CoV-2 NC and S test results, we estimated the seroprevalence in a sample of Dutch blood donors and distinguished between infection and vaccine induced immunity. Using the Wantai and both Elecsys® assays, we classified half of the donors in the cross-sectional study as not exposed to SARS-CoV-2, approximately 32% as vaccinated and almost 17% as has been infected with SARS-CoV-2 by May 2021 (of note, only individuals aged >50 years old were eligible for vaccination at that time [14]). We could not distinguish between infected persons with or without subsequent vaccination. The results were comparable when using the Wantai Ab ELISA for anti-S testing instead of the Elecsys® S assay.

The specificity and sensitivity of SARS-CoV-2 antibody assays is generally studied and validated shortly after infection and in individuals with PCR-proven infection. The Elecsys® NC assay and the Elecsys® S assay were shown to be highly sensitive and specific in this manner [15–18]. The immune response against SARS-CoV-2 may be less pronounced in COVID patients who experienced mild symptoms. Because of limited test capacity early in the pandemic, patients with mild symptoms were generally not tested and may be underrepresented in early validation studies [19]. In addition, it was shown that waning antibody titers may result in false negative results in patients, already few months after infection [7]. The rate of waning may strongly depend on the assay being used [9]. A strength of this study is that we studied the ‘average’ immune response and the rate of waning of antibody titers in a cohort that represents the average population and that is not biased towards PCR-tested persons and patients with severe symptoms.

Follow-up of blood donors that were identified in our first national serosurveillance study provided an excellent opportunity to monitor the long term humoral immune response in time. Few studies have reported on the long term-response. We show that the majority of donors who experienced infection in the first half of 2020 were still antibody positive two years later in the Elecsys NC assay. Some seroreversions were noticed, however, and the use of a reduced cutoff resulted in a more sustained sensitivity longer after infection. Approximately 10% more donors remained positive for anti-NC after a 2-year period if the reduced cutoff was employed. This improved sensitivity possibly results in a slightly reduced specificity, but it should be noted that the proportion of individuals in the COI range between 0.3 and 1 prior to the COVID pandemic was very low [9]. The well-sustained anti-NC response contrasts with observations made in Brazil with another commercial anti-NC antibody assay that showed very rapid waning after infection [7]. The difference may be related to the different test formats; the Elecsys® assay is an antigen sandwich assay which may provide better sensitivity than an indirect IgG EIA format.

Our cross-sectional study contained a comparison between the Wantai and Elecsys® S assay. We observed very similar performance and did not observe a significantly lower specificity of the Wantai assay compared with the Elecsys® S assay, as reported in a large-scale comparison of commercial anti-SARS-CoV-2 tests [9]. In this study by Stone *et al*. the specificity of the Wantai assay was lower (98.6%) than the >99.5% of the Elecsys® assays, testing a large pre-pandemic panel of samples. We previously estimated the specificity of the Wantai test to be 99.6% and have no good explanation for the difference [11]. Since our lab performed the Wantai testing for the study from Stone *et al*. it seems more likely to be related to the samples than to the testing.

A limitation of this study is that we had no data on whether individuals showed signs of infection, were PCR tested, or received vaccination. This means we may have missed donors who did contract SARS-CoV-2 or received vaccination but did not develop sufficient antibody levels to be detected. Also, due to waning of NC antibodies, some donors might be misclassified as vaccinated instead of infected. Another limitation is that if we want to generalize the donor data to the general Dutch population a few factors should be taken into consideration. First of all, blood donors undergo pre-donation screening and are therefore generally more healthy than the general population, a selection bias often referred to as the healthy donor effect (HDE) [20]. Individuals with chronic or acute disease or transfusion transmissible infections, and individuals older than 80 years are not allowed to donate. Additionally, there may be donors who chose not to donate during the pandemic because of fear of infection during the donation process or objections against the infection preventive measures taken by the blood bank during the pandemic (physical distancing, wearing a face mask).

## Conclusion

In conclusion, we show that the Elecsys® S and Elecsys® NC antibody assays can be reliably used to differentiate between vaccination and infection. Additionally, both assays have a sufficient long-term performance, with antibody levels still detectable 1–2 years after infection in the majority of donors. Further studies are required to determine whether a sustained anti-NC response also occurs in donors who have been vaccinated before infection, or in donors who were infected with later variants of SARS-CoV-2.

## Supporting information

S1 Table Results from sample selection: Age and sex distribution relative to population. (DOCX)

S2 Table Results from sample selection: Regional distribution. (DOCX)

S3 Table Distinguishing between vaccinated and infected donors using a different cut-off value for the Elecsys® NC assay. (DOCX)

S1 Fig Results of repeat testing of samples with an OD/CO ratio>0.25 in the Wantai test using either the kit lot used for initial screening (left) or a recently produced kit lot (right). The solid line shows perfect agreement (y = x).
(DOCX)

S1 Data (CSV)

S2 Data (CSV)
