# Supplementary material for: The Elecsys® Anti-SARS-CoV-2 and Elecsys® Anti-SARS-CoV-2 S antibody assays: Differentiating between vaccination and infection, and assessing long-term performance
Source: PLoS One. 2024 Jul 18;19(7):e0305613. doi: 10.1371/journal.pone.0305613 (PMC11257240; doi:10.1371/journal.pone.0305613)
Supplement: S1 Table — (DOCX) [file pone.0305613.s001.docx]

**S1 Table. Results from sample selection: Age and sex distribution relative to population.**

|  | **Selected donations** | | | **All donations (May 17-May 21, 2021)** | | | **Dutch population** | | |
| --- | --- | --- | --- | --- | --- | --- | --- | --- | --- |
| Age group | No. of donations | % male | % age cat | No. of donations | % male | % age cat | No of inhabitants | % male | % age cat |
| 18-30 | 286 | 51.4 | 13.1 | 4352 | 36.9 | 23.8 | 2174645* | 49.3 | 17.3 |
| 31-40 | 344 | 50.6 | 15.8 | 2488 | 49.5 | 13.6 | 2077670 | 49.8 | 16.5 |
| 41-50 | 412 | 51.9 | 18.9 | 3178 | 47.0 | 17.4 | 2306840 | 50.1 | 18.3 |
| 51-60 | 424 | 48.8 | 19.4 | 4066 | 54.0 | 22.3 | 2491405 | 49.8 | 19.8 |
| 61-70 | 491 | 47.7 | 22.5 | 3666 | 63.8 | 20.1 | 2079390 | 50.3 | 16.5 |
| >70 | 227 | 69.6 | 10.4 | 502 | 71.5 | 2.8 | 1460450 | 52.2 | 11.6 |
| Total | 2184 | 51.9 |  | 18252 | 50.6 |  | 12590400 | 50.1 |  |

*: age 20-30 (no details known about 18-20)
