# Supplementary material for: The Elecsys® Anti-SARS-CoV-2 and Elecsys® Anti-SARS-CoV-2 S antibody assays: Differentiating between vaccination and infection, and assessing long-term performance
Source: PLoS One. 2024 Jul 18;19(7):e0305613. doi: 10.1371/journal.pone.0305613 (PMC11257240; doi:10.1371/journal.pone.0305613)
Supplement: S2 Table — (DOCX) [file pone.0305613.s002.docx]

**S2 Table. Results from sample selection: Regional distribution.**

|  |  | Selected donations | | | All donations (May 17-May 21) | | |
| --- | --- | --- | --- | --- | --- | --- | --- |
| Municipal Health Region | Population | No. Tested | % male | Tested / 10000 | Donations | % male | Donations/100000 |
| Amsterdam | 1046450 (6.1%) | 107 (4.9%) | 57.9 | 10.2 | 563 (3.1%) | 50.8 | 53.8 |
| Brabant-Zuidoost | 766745 (4.5%) | 107 (4.9%) | 43.0 | 14.0 | 881 (4.8%) | 47.7 | 114.9 |
| Drenthe | 492005 (2.9%) | 69 (3.2%) | 43.5 | 14.0 | 384 (2.1%) | 46.1 | 78.0 |
| Flevoland | 411675 (2.4%) | 22 (1%) | 63.6 | 5.3 | 65 (0.4%) | 66.2 | 15.8 |
| Fryslân | 647260 (3.8%) | 91 (4.2%) | 58.2 | 14.1 | 909 (5%) | 49.8 | 140.4 |
| Gelderland Midden | 685150 (4%) | 62 (2.8%) | 50.0 | 9.0 | 694 (3.8%) | 53.0 | 101.3 |
| Gelderland-Zuid | 553630 (3.2%) | 88 (4%) | 51.1 | 15.9 | 717 (3.9%) | 48.8 | 129.5 |
| Gooi en Vechtstreek | 252975 (1.5%) | 14 (0.6%) | 28.6 | 5.5 | 177 (1%) | 47.5 | 70.0 |
| Groningen | 582940 (3.4%) | 79 (3.6%) | 54.4 | 13.6 | 696 (3.8%) | 43.0 | 119.4 |
| Haaglanden | 1093180 (6.4%) | 125 (5.7%) | 51.2 | 11.4 | 843 (4.6%) | 55.2 | 77.1 |
| Hart voor Brabant | 1058365 (6.2%) | 124 (5.7%) | 51.6 | 11.7 | 1372 (7.5%) | 47.2 | 129.6 |
| Hollands Midden | 794340 (4.6%) | 101 (4.6%) | 55.4 | 12.7 | 1097 (6%) | 51.4 | 138.1 |
| Hollands Noorden | 655200 (3.8%) | 90 (4.1%) | 48.9 | 13.7 | 777 (4.3%) | 50.5 | 118.6 |
| IJsselland | 523560 (3%) | 61 (2.8%) | 55.7 | 11.7 | 1007 (5.5%) | 52.6 | 192.3 |
| Kennemerland | 541620 (3.2%) | 75 (3.4%) | 56.0 | 13.8 | 420 (2.3%) | 49.0 | 77.5 |
| Noord en Midden Limburg | 518155 (3%) | 76 (3.5%) | 48.7 | 14.7 | 527 (2.9%) | 55.4 | 101.7 |
| Noord en Oost Gelderland | 821125 (4.8%) | 123 (5.6%) | 48.0 | 15.0 | 751 (4.1%) | 51.3 | 91.5 |
| Regio Twente | 627745 (3.7%) | 74 (3.4%) | 60.8 | 11.8 | 773 (4.2%) | 49.9 | 123.1 |
| Regio Utrecht | 1295440 (7.5%) | 214 (9.8%) | 50.5 | 16.5 | 1788 (9.8%) | 50.7 | 138.0 |
| Rotterdam-Rijnmond | 1303205 (7.6%) | 175 (8%) | 54.3 | 13.4 | 988 (5.4%) | 52.7 | 75.8 |
| West-Brabant | 702870 (4.1%) | 85 (3.9%) | 49.4 | 12.1 | 647 (3.5%) | 51.6 | 92.1 |
| Zaanstreek/Waterland | 334740 (1.9%) | 37 (1.7%) | 51.4 | 11.1 | 417 (2.3%) | 49.9 | 124.6 |
| Zeeland | 382325 (2.2%) | 26 (1.2%) | 53.8 | 6.8 | 387 (2.1%) | 48.6 | 101.2 |
| Zuid Holland-Zuid | 490180 (2.9%) | 64 (2.9%) | 56.3 | 13.1 | 550 (3%) | 51.3 | 112.2 |
| Zuid-Limburg | 599000 (3.5%) | 83 (3.8%) | 49.4 | 13.9 | 693 (3.8%) | 53.8 | 115.7 |
| N.a. (Foreigner, Unknown) | n.a. | 12 (0.5%) | 50.0 | n.a. | 129 (0.7%) | 51.9 | n.a. |
