# Supplementary material for: The Elecsys® Anti-SARS-CoV-2 and Elecsys® Anti-SARS-CoV-2 S antibody assays: Differentiating between vaccination and infection, and assessing long-term performance
Source: PLoS One. 2024 Jul 18;19(7):e0305613. doi: 10.1371/journal.pone.0305613 (PMC11257240; doi:10.1371/journal.pone.0305613)
Supplement: S3 Table — (DOCX) [file pone.0305613.s003.docx]

**S3 Table. Distinguishing between vaccinated and infected donors using a different cut-off value for the Elecsys® NC assay.**

| **Decreased cutoff (<0.3)** | **S Neg** | **S Pos** | **Total** |
| --- | --- | --- | --- |
| NC Neg | 1095 | 689 | 1784 |
| NC Pos | 10 | 388 | 398 |
| Total | 1105 | 1077 | 2182 |
| **Standard cutoff (<1)** |  |  |  |
| NC Neg | 1101 | 710 | 1811 |
| NC Pos | 4 | 367 | 371 |
| Total | 1105 | 1077 | 2182 |
