# Supplementary material for: The Elecsys® Anti-SARS-CoV-2 and Elecsys® Anti-SARS-CoV-2 S antibody assays: Differentiating between vaccination and infection, and assessing long-term performance
Source: PLoS One. 2024 Jul 18;19(7):e0305613. doi: 10.1371/journal.pone.0305613 (PMC11257240; doi:10.1371/journal.pone.0305613)
Supplement: S1 Fig — Results of repeat testing of samples with an OD/CO ratio>0.25 in the Wantai test using either the kit lot used for initial screening (left) or a recently produced kit lot (right). The solid line shows perfect agreement (y = x). (DOCX) [file pone.0305613.s004.docx]

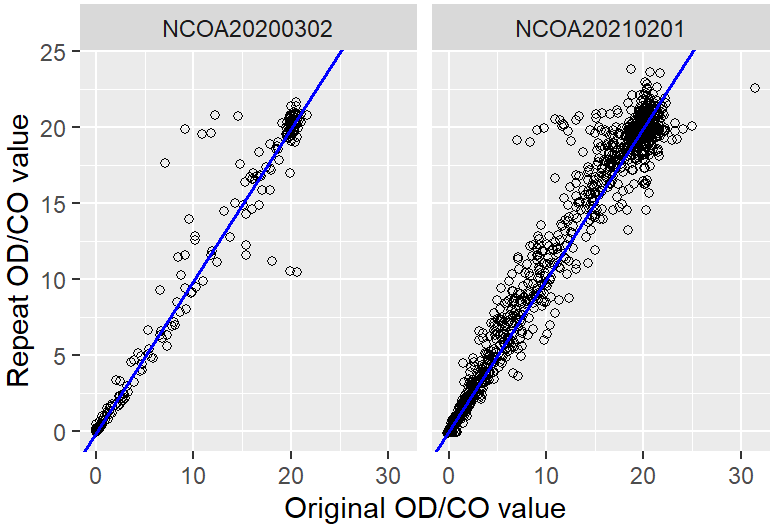


**S1 Figure. Results of repeat testing of samples with an OD/CO ratio>0.25 in the Wantai test using either the kit lot used for initial screening (left) or a recently produced kit lot (right). The solid line shows perfect agreement (y=x).**
